# Knowledge, attitudes, and practices of healthcare providers in pre-hospital care in Nepal

**DOI:** 10.3389/fpubh.2025.1623868

**Published:** 2025-07-25

**Authors:** Pavan Kumar Sah, Wenyi Lai, Neelam Gupta, Bimal Singh Bist, Sanjib Gautam, Khem Raj Sapkota, Bipin Koirala, Ileana Kong Zhuo, Morlai Sesay, Ying Guan

**Affiliations:** ^1^Department of Biostatistics, School of Public Health, Southern Medical University, Guangzhou, China; ^2^Department of Pediatrics, Paropakar Maternity and Women's Hospital, Kathmandu, Nepal; ^3^Department of Health Education, Graduate School of Education, Tribhuvan University, Kathmandu, Nepal; ^4^Nepal College of Information Technology, Lalitpur, Nepal; ^5^School of International Education, Southern Medical University, Guangzhou, China

**Keywords:** emergency medical services, Sustainable Development Goals, healthcare workers, pre-hospital system, Nepal, KAP survey

## Abstract

**Introduction:**

Pre-hospital emergency care is crucial for improving patient outcomes, especially in low- and middle-income countries (LMICs) where trauma is a leading cause of death. In Nepal, inadequate pre-hospital care contributes to approximately 16,600 preventable deaths annually. This study assessed the knowledge, attitudes, and practices (KAP) of healthcare providers in pre-hospital care to identify factors influencing their preparedness.

**Methods:**

A quantitative, descriptive cross-sectional design was employed, utilizing a 35-item questionnaire based on a 5-point Likert scale. Data were collected via an online survey (Google Forms) from 517 healthcare providers (doctors, nurses, and paramedics) across 16 hubs and 76 satellite hospitals in Nepal’s seven provinces based on a systematic randomization technique. Data were described with median and interquartile range. Nonparametric analysis, rank Spearman’s rank correlation, and ordinal regression were used to analyze the data.

**Results:**

The study revealed that 62% of providers had good knowledge, 66% exhibited positive attitudes, but only 25% demonstrated good practice. Significant variations were observed by gender, profession, and workplace, with males, doctors, and private hospital providers scoring higher in knowledge and practice. Moderate correlations were found between knowledge, attitude, and practice (rs = 0.420–0.562, *p* < 0.001). Ordinal logistic regression indicated significant associations between demographic factors and KAP levels.

**Conclusion:**

Despite good knowledge and positive attitudes, only 25% demonstrated good practice, indicating practical implementation of pre-hospital care remains suboptimal. Targeted training programs, simulation-based learning, and continuous professional development will be needed to bridge the gap between knowledge and practice.

## Introduction

Pre-hospital emergency care is a critical component of the emergency medical services (EMS) system, providing immediate medical assistance to patients at the scene of an incident or injury before they reach a healthcare facility. Timely and appropriate pre-hospital care can significantly influence patient outcomes, reducing their morbidity and mortality rates (1). Globally, trauma is a leading cause of death and disability, with over 5.8 million deaths annually attributed to injuries, the majority of which occur in low- and middle-income countries (LMICs) where pre-hospital care systems are often underdeveloped (2, 3). In Nepal, approximately 16,600 preventable deaths occur annually due to inadequate pre-hospital care, with trauma being a major contributor (4).

In LMICs, the lack of trained personnel, inadequate infrastructure, geographical barriers, and cultural factors exacerbate the challenges of pre-hospital care (3, 5). In Nepal, these challenges are compounded by a fragmented EMS system, limited ambulance services, and difficult geographical conditions (6).

Healthcare providers play a central role in pre-hospital care, as they are often the first point of contact for patients in emergencies. Their ability to perform triage, provide immediate interventions, and stabilize patients during transport is essential to improving outcomes. However, the effectiveness of these interventions largely depends on the providers’ knowledge, attitudes, and practices (KAP) about pre-hospital emergency care. Studies from LMICs suggested that gaps in training and preparedness among healthcare providers contributed significantly to poor outcomes in emergency scenarios (7, 8). However, we know little about the KAP of healthcare providers in Nepal’s pre-hospital care system.

Strengthening pre-hospital care systems is directly aligned with the Sustainable Development Goals (SDG), particularly SDG 3.6, which aims to reduce global road traffic deaths and injuries, and SDG 3.8, which promotes universal health coverage and access to quality essential health services. According to the WHO’s 2023 report “Strengthening Emergency, Critical and Operative Care Services,” integrating EMS into national health strategies is essential for reducing avoidable deaths and achieving equitable health outcomes in LMICs (9). The report emphasizes the need for improved EMS governance, standardized training of providers, and context-specific investment in frontline healthcare systems—including pre-hospital services.

Our study aims to assess the KAP of healthcare providers in Nepal’s pre-hospital care system and identify factors influencing their preparedness. By understanding the strengths and weaknesses in their preparedness, this research seeks to guide targeted interventions to improve training, policies, and resource allocation. Additionally, it provides actionable recommendations for policymakers and stakeholders to build a more responsive and effective EMS framework.

## Materials and methods

### Study design and setting

A cross-sectional design was employed, utilizing an online survey via Google Forms to assess the KAP of healthcare providers in pre-hospital care in Nepal. The study included 16 out of 25 hub hospitals and 76 out of 150 satellite hospitals across all seven provinces, selected through a systematic proportional sampling method to ensure representation based on healthcare facility availability and population size. Both urban and rural settings were incorporated to enhance geographical diversity.

### Study populations and inclusion and exclusion criteria

The study population included healthcare providers in pre-hospital care including doctors, nurses, and paramedics who worked in the emergency units of hub and satellite hospitals in Nepal. All healthcare providers who were actively working in emergency units with experience of more than 6 months in emergency care during data collection were included. The healthcare providers who refused to give their consent to participate in the study were excluded.

### Sample size estimation

The sample size was estimated using the formula (Equation 1) for a cross-sectional study, assuming a proportion (p) of 0.5, a margin of error (ɛ) of 0.05, and a z-score of 1.96 at a 95% confidence interval. The estimated sample size was 384, with an additional 10% non-response rate considered, resulting in a final sample size of 423.


(1) $$n=\frac{z^{2}p(1-p)}{\varepsilon^{2}}$$

### Sampling method

**Figure 1:**
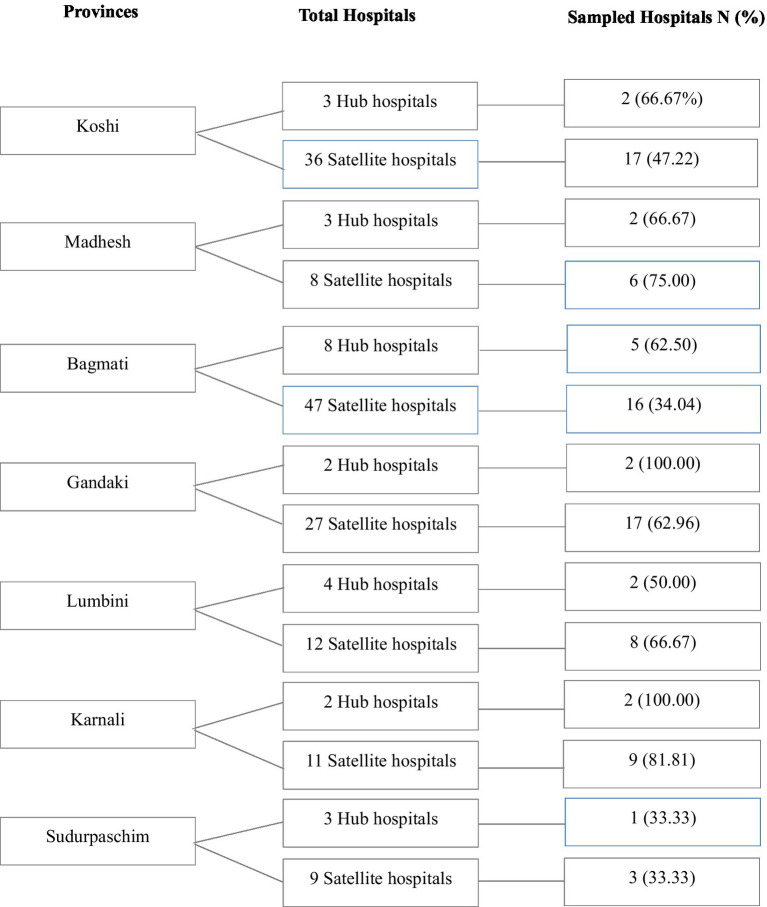
Distributions of total and sampled hub and satellite hospitals across provinces in Nepal.

A multistage, systematic random sampling approach was employed. From a national list of 25 hub and 150 satellite hospitals, 16 hub (64%) and 76 satellite hospitals (51%) were proportionally selected, representing 52.6% of eligible facilities. Hospitals selections were sampled based on geographical distribution, population size and facility availability across provinces. Within each hospital, doctors, nurses, and paramedics were randomly selected from emergency unit duty rosters. Although the estimated sample size was 423, a total of 517 responses were collected (130 from hub and 387 from satellite hospitals). While voluntary online participation may introduce non-response bias, broad representation across regions and professions enhances the study’s validity (Figure 1).

### Study variables

In this study, the dependent variables were KAP scores of healthcare workers related to pre-hospital care, assessed using a 35-item questionnaire based on a 5-point Likert scale (1 = strongly disagree, 5 = strongly agree). The questionnaire included 15 questions on knowledge, 10 on attitude, and 10 on practice. Scores were categorized into three levels: poor, average, and good based on a predefined percentage method of categorization (10, 11). Specifically, knowledge scores were categorized as poor (15–35), average (36–55), and good (56–75); attitude and practice scores were categorized as poor (10–23), average (24–36), and good (37–50). The eight independent variables were included such as gender, age, marital status, profession, years of experience, type of hospital, province of practice, and geographical region.

### Data collection tool and validation

The questionnaire, adapted from a previous study in Ethiopia (1), included five sections: Consent Form, Demographic Information, and Knowledge, Attitudes, and Practices assessments. A panel of nine experts with 13–23 years of experience validated the tool, and pre-testing among 42 participants yielded a Cronbach’s alpha of 0.947, and a Kaiser-Meyer-Olkin (KMO) value of 0.946, calculated using SPSS version 26, confirming high internal consistency and sample adequacy. Data were collected electronically via email, Viber, and WhatsApp. The questionnaire took approximately 10–15 min to complete, as observed during pre-testing.

### Statistical analysis

Data were analyzed using SPSS version 26. Descriptive statistics summarized demographic characteristics and KAP scores. Non-parametric tests were used due to non-normal data distribution: Mann–Whitney U for two-group comparisons, Kruskal-Wallis H for multi-group comparisons, and Spearman’s Rank Correlation and ordinal regression for assessing relationships among variables. Chi-Square tests compared categorical variables. Statistical significance was set at *α* = 0.05, and *p* < 0.05 was statistically significant.

### Ethical considerations

Ethical approval was obtained from the Nepal Health Research Council (Ref. No: 417/2024). Informed consent was obtained from all participants, and confidentiality was maintained through anonymized data storage.

## Results

### Demographic characteristics

As Table 1 showed the study sample included 517 participants, predominantly male (69.6%) and aged 20–30 years (52.6%). The majority were married (55.7%), with doctors forming the largest professional group (46.4%). Most participants had 1–5 years of experience (40.4%) and worked in public hospitals (77.6%), particularly in satellite hospitals (74.9%). Geographically, participants were spread across various provinces, with the Bagmati and Koshi provinces contributing the most (50.0%). The majority practiced in the Hill region (51.5%), followed by the Terai (34.2%) and the Mountain region (14.3%).

**Table 1:** Demographic characteristics of participants.

Variables		Frequency	Percentage
Gender	Male	360	69.6
	Female	157	30.4
Age (Years)	20–30	272	52.6
	31–40	165	31.9
	> 40	80	15.5
Marital status	Single	229	44.3
	Married	288	55.7
Profession	Doctor	240	46.4
	Nurse	93	18.0
	Paramedic	184	35.6
Experience in healthcare (Years)	<1	57	11.0
	1–5	209	40.4
	6–10	122	23.6
	11–15	60	11.6
	> 15	69	13.4
Current workplace	Public Hospital	401	77.6
	Private Hospital	116	22.4
Type of Hospital	Hub Hospital	130	25.1
	Satellite Hospital	387	74.9
Provinces	Koshi	130	25.1
	Madhesh	49	9.5
	Bagmati	146	28.2
	Gandaki	77	14.9
	Lumbini	44	8.5
	Karnali	35	6.8
	Sudurpaschim	36	7.0
Geographical region	Terai	177	34.2
	Hill	266	51.5
	Mountain	74	14.3

### Knowledge, attitude, and practice levels

**Figure 2:**
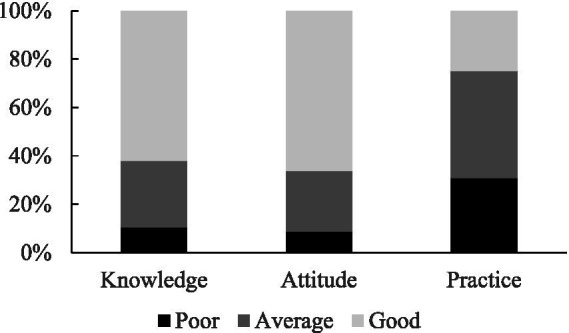
The percentage of three levels of KAP in healthcare providers in Nepal’s pre-hospital care system.

The KAP scores were categorized into three levels: poor, average, and good. The results revealed that 62.1% of healthcare providers demonstrated good knowledge, while 10.4% had poor knowledge. Similarly, 66.3% exhibited a good attitude, with 8.7% showing a poor attitude. However, only 25.0% demonstrated good practice, while 30.8% had poor practice, indicating a significant gap between knowledge and practice (Figure 2).

### Demographic factors influencing KAP scores

Knowledge levels varied significantly across demographic groups (Table 2). Male and younger healthcare providers had higher median knowledge scores compared to females and older ones (*p* = 0.001 and 0.002). Doctors exhibited the highest median knowledge score (62), followed by paramedics (58) and nurses (55, *p* < 0.001). Healthcare providers in private hospitals had significantly higher knowledge scores (62) compared to those in public hospitals (59, *p* = 0.001). Participants from the Hill region had the highest median knowledge score (61), while those from the Terai region scored the lowest (57, *p* = 0.020).

**Table 2:** Comparison of knowledge among healthcare providers based on demographic characteristics.

Variables		Knowledge		Attitude		Practice	
		Md (IQR)	Statistical Test (*p*-value)	Md (IQR)	Statistical Test (*p*-value)	Md (IQR)	Statistical Test (*p*-value)
Gender	Male	60 (14.7)	U = −3.257 (0.001)	39 (7.0)	U = −0.228 (0.820)	31 (15.0)	U = −3.700 (<0.001)
	Female	55 (16.0)		38 (8.0)		27 (14.0)	
Marital status	Single	58 (14.0)	*U* = −1.634 (0.100)	39 (7.0)	*U* = −0.877 (0.380)	29 (13.0)	*U* = −0.653 (0.510)
	Married	60 (17.0)		38 (8.0)		30 (16.0)	
Age group	20–30	58 (14.0)	*H* = 12.245 (0.002)	40 (7.0)	*H* = 1.75 (0.556)	30 (15.0)	*H* = 5.219 (0.074)
	31–40	61 (15.5)		39 (7.0)		30 (15.0)	
	>41	63 (16.0)		38 (8.0)		26 (15.0)	
Profession	Doctor	62 (13.0)	*H* = 21.866 (<0.001)	39 (7.0)	*H* = 1.303 (0.510)	31 (15.0)	*H* = 0.521 (0.002)
	Nurse	55 (17.0)		39 (6.0)		26 (13.5)	
	Paramedic	58 (15.5)		39 (8.0)		29 (16.0)	
Experience in healthcare (Years)	<1	56 (14.5)	*H* = 10.268 (0.040)	39 (5.5)	*H* = 2.215 (0.700)	27 (12.5)	*H* = 8.208 (0.090)
	1–5	59 (14.0)		39 (8.0)		30 (15.0)	
	6–10	61 (15.3)		39 (6.3)		30 (16.0)	
	11–15	62 (11.0)		38 (7.8)		29 (13.0)	
	>15	60 (23.0)		38 (8.0)		25 (15.5)	
Current workplace	Public Hospital	59 (16.0)	*U* = −3.269 (0.001)	38 (8.0)	*U* = −2.303 (0.021)	29 (14.0)	*U* = −3.864 (<0.001)
	Private Hospital	62 (12.8)		40 (7.0)		33 (13.8)	
Type of hospital	Hub Hospital	61 (13.3)	*U* = −2.310 (0.020)	40 (8.0)	*U* = −0.794 (0.430)	30 (16.3)	*U* = −1.018 (0.300)
	Satellite Hospital	59 (15.0)		38 (7.0)		29 (15.0)	
Province	Koshi Province	55 (17.3)	*H* = 33.016 (<0.001)	38 (8.0)	*H* = 11.322 (0.080)	29 (15.0)	*H* = 13.027 (0.040)
	Madhesh Province	56 (13.0)		39 (8.5)		29 (17.0)	
	Bagmati Province	63 (14.0)		40 (7.0)		32 (15.0)	
	Gandaki Province	58 (14.5)		38 (7.5)		28 (14.0)	
	Lumbini Province	60 (17.3)		37.5 (8.0)		27 (14.0)	
	Karnali Province	63 (10.0)		41 (11.0)		31 (17.0)	
	Sudurpaschim Province	59 (14.5)		39 (7.8)		26 (13.0)	
Geographical regions	Terai	57 (14.0)	*H* = 8.478 (0.020)	38 (7.0)	*H* = 4.464 (0.100)	28 (14.5)	*H* = 3.858 (0.140)
	Hill	61 (16.0)		39(7.3)		30 (15.0)	
	Mountain	60 (15.0)		39(6.3)		30(11.8)	

Md, median; IQR, interquartile range; U, Man Whitney test; H, Kruskal-Wallis test.

Attitude levels also showed significant differences among different demographic factors. Healthcare providers in private hospitals reported higher median attitude scores (40) compared to those in public hospitals (38, *p* = 0.021). Participants from Bagmati Province had the highest proportion of good attitude levels (31.5%), while Lumbini Province had the lowest (7.6%, *p* = 0.037).

Practice levels were significantly influenced by gender, profession, and workplace. Males had higher median practice scores (31) compared to females (27, *p* < 0.001). Doctors reported the highest median practice score (31), followed by paramedics (29) and nurses (26, *p* = 0.002). Participants in private hospitals had higher practice scores (33) compared to those in public hospitals (29, p < 0.001). Healthcare providers from Bagmati Province had the highest median practice score (32), while those from Sudurpaschim Province scored the lowest (26, *p* = 0.040).

### Comparison of KAP levels among participants based on demographic characteristics

We organized the significant results in Table 3. The result revealed significant variations in knowledge, attitudes, and practices (KAP) levels across demographic factors.

**Table 3:** Comparison of knowledge levels among healthcare providers based on demographic characteristics.

	Variables		Poor *n* (%)	Average *n* (%)	Good *n* (%)	*χ* ^2^	*p*-value
Knowledge	Gender	Male	36 (66.7%)	81 (57%)	243 (75.7%)	16.460	<0.001
		Female	18 (33.3%)	61 (43%)	78 (24.3%)		
	Age (Years)	20–30	25 (46.3%)	90 (63.4%)	157 (52.6%)	10.808	0.029
		31–40	21 (38.9%)	38 (26.8%)	106(33%)		
		> 40	8 (14.8%)	14 (9.9%)	58(18.1%)		
	Profession	Doctor	19 (35.2%)	51 (35.9%)	170(53%)	22.162	<0.001
		Nurse	9 (16.7%)	40 (28.2%)	44 (47.3%)		
		Paramedic	26 (48.1%)	51 (35.9%)	107 (33.3%)		
	Current workplace	Public Hospital	44 (81.5%)	124 (87.3%)	233 (72.6%)	12.820	0.002
		Private Hospital	10 (18.5%)	18 (12.7%)	88 (27.4%)		
	Province	Koshi	22 (40.7%)	45 (31.7%)	63 (19.6%)	33.778	0.001
		Madhesh	6 (11.1%)	16 (11.3%)	27 (8.4%)		
		Bagmati	14 (25.9%)	24 (16.9%)	108 (33.6%)		
		Gandaki	6 (11.1%)	27 (19%)	44 (13.7%)		
		Lumbini	0 (0%)	15 (10.6%)	29 (9.0%)		
		Karnali	2 (3.7%)	6 (4.2%)	27 (8.4%)		
		Sudurpaschim	4 (7.4%)	9 (1.7%)	23 (4.4%)		
Attitude	Province	Koshi	16 (35.6%)	39 (30.2%)	75 (21.9%)	22.039	0.037
		Madhesh	4 (8.9%)	11 (8.5%)	34 (9.9%)		
		Bagmati	12 (26.7%)	26 (20.2%)	108 (31.5%)		
		Gandaki	6 (13.3%)	20 (15.5%)	51 (14.9%)		
		Lumbini	1 (2.2%)	17 (13.2%)	26 (7.6%)		
		Karnali	1 (2.2%)	12 (9.3%)	22 (6.4%)		
		Sudurpaschim	15 (11.1%)	4 (3.1%)	26 (7.0%)		
Practice	Gender	Male	95 (59.7%)	164 (71.6%)	101 (78.3%)	12.349	0.002
		Female	64 (40.3%)	65 (28.4%)	28 (21.7%)		
	Current workplace	Public Hospital	135 (84.9%)	180 (78.6%)	86 (66.7%)	13.869	0.001
		Private Hospital	24 (15.1%)	49 (21.4%)	43 (33.3%)		
	Experience in healthcare (Years)	<1	20 (12.6%)	28 (12.2%)	9 (7.0%)	16.705	0.033
		1–5	55 (34.6%)	95 (41.5%)	59 (45.7%)		
		6–10	35 (22.0%)	49 (21.4%)	38(29.5%)		
		11–15	18 (11.3%)	32 (14.0%)	10 (7.7%)		
		> 15	31 (19.5%)	25 (10.9%)	13 (10.1%)		

*χ*^2^, chi square test.

Significant associations with knowledge levels were observed for gender, age, profession, workplace and province of practice. Males, doctors, and providers in private hospitals exhibited higher knowledge levels, with those from Bagmati province also showing superior knowledge. Additionally, the 20–30 age group had a significant prevalence in the average knowledge category.

In contrast, attitude levels showed no significant differences across most demographics, except for the province of practice, where notable variations were observed (*χ*^2^ = 22.039, *p* = 0.037).

For practice levels, significant associations were found with gender, workplace, and years of healthcare experience, with males, private hospital employees, and those with 6–10 years of experience demonstrating better practices. These findings highlight the influence of demographic factors on KAP levels among healthcare providers.

### Correlations and regression analysis

Correlation analysis revealed moderate positive correlations among knowledge, attitude and practice with r_s_ ranging from 0.420 to 0.562 (*p* < 0.001) (Table 4). Ordinal logistic regression analysis (Table 5) revealed significant associations between demographic factors and KAP levels. Males and doctors had higher knowledge levels. Healthcare providers in private hospitals also showed greater knowledge. Males and those in private hospitals exhibited more positive attitudes. Similarly, males and healthcare providers in private hospitals engaged in better practices.

**Table 4:** Correlation analyses between knowledge, attitude, and practice.

Variables	Knowledge	Attitude	Practice
Knowledge	1.000	–	–
Attitude	0.562**	1.000	–
Practice	0.420**	0.431**	1.000

**p < 0.01 with 2-tailed test.

**Table 5:** Ordinal logistic regression among KAP and demographic characteristics.

DV	IV		Estimate	SE	Wald	df	*p*-value	95% CI	
								Lower	Upper
Knowledge	Gender	Male	0.421	0.210	4.007	1	0.045	0.009	0.834
	Profession	Doctor	0.426	0.189	5.084	1	0.024	0.056	0.797
		Nurse	−0.090	0.253	0.126	1	0.723	−0.586	0.407
		Paramedic	Ref						
	Hospital	Private	0.691	0.246	7.905	1	0.005	0.209	1.173
		Public	Ref						
Attitude	Gender	Male	0.463	0.172	7.270	1	0.007	0.126	0.800
	Hospital	Private	0.500	0.162	9.528	1	0.002	0.182	0.817
		Public	Ref						
	Geographical region	Terai	−0.496	0.245	4.103	1	0.043	−0.976	−0.016
		Hill	−0.180	0.197	0.838	1	0.360	−0.566	0.206
		Mountain	Ref						
Practice	Gender	Male	0.305	0.159	3.654	1	0.046	−0.008	0.617
	Hospital	Private	0.463	0.166	7.799	1	0.005	0.138	0.787
		Public	Ref						

DV, dependent variable; IV, independent variable; SE, standard error; CI, confidence interval.

## Discussion

### Knowledge, attitude and practice gaps

The study revealed that a majority of healthcare providers (62.1%) demonstrated good knowledge of pre-hospital care in Nepal. This finding aligns with a study conducted in Sri Lanka, where most healthcare workers exhibited above-average knowledge at the basic level of pre-hospital care (12). This positive outcome in Nepal is likely due to recent government initiatives such as the Health Sector Implementation Plan (2016–21) and the 2021 Emergency Care System Assessment, which prioritized emergency medical services (EMS) and outlined 39 key priorities to boost emergency care quality. The first national conference on pre-hospital care also emphasized the importance of universal access to quality care, raising awareness. Moreover, an increased emphasis on emergency care training, especially in urban areas, along with better resources and systems, has contributed to improved knowledge among providers (13). However, this contrasts with findings from studies in Ethiopia, Indonesia, India, and Nepal, where knowledge levels were notably lower. For instance, in Ethiopia, less than half (42.9%) of nurses had good knowledge of pre-hospital emergency care (1). Similarly, a study in Indonesia reported low knowledge levels among healthcare providers, particularly in community health settings (14). In India, research highlighted inadequate knowledge related to pre-hospital and emergency care among healthcare providers (15). Studies in Nepal also found that knowledge about basic life support and pre-hospital care was insufficient among healthcare workers (16, 17). Despite these regional variations, the current study identified gaps in knowledge, with 27.5% of participants demonstrating average knowledge and 10.4% exhibiting poor knowledge. This underscores the need for enhanced training in emergency response protocols and patient assessment techniques.

Despite relatively good knowledge and positive attitudes, the study found that actual practice in pre-hospital care was suboptimal. Only 25% of healthcare providers demonstrated good practice, while 44.3% had average practice, and 30.8% exhibited poor practice. This gap between knowledge, attitude, and practice was documented in other studies, emphasizing that theoretical knowledge does not always translate into effective implementation (1, 14, 15). The moderate positive correlations between knowledge, attitudes, and practices (rs = 0.420–0.562, *p* < 0.001) suggest that improving knowledge and fostering positive attitudes could enhance practice behaviors. However, the relatively lower correlation between knowledge and practice (rs = 0.420) indicates the need for targeted interventions, such as hands-on training, simulation-based learning, and continuous professional development programs, to bridge this gap.

### Demographic and institutional variations

The study also identified significant variations in knowledge levels based on demographic factors such as gender, profession, years of experience, and type of hospital. Male healthcare providers had significantly higher knowledge scores than their female counterparts, a finding consistent with studies in Ethiopia, where male nurses exhibited better knowledge, potentially due to greater exposure to emergency cases (1). In Nepal, the higher number of male healthcare professionals and their greater involvement in treatment and pre-hospital emergency care may account for this disparity. Additionally, societal expectations and gender roles may influence access to training opportunities, with males potentially having greater access to specialized pre-hospital care programs. Future research should explore these factors in detail to better understand the underlying causes of gender disparities in knowledge levels.

Doctors demonstrated the highest knowledge levels, followed by paramedics and nurses, a finding consistent with studies in India and Nepal (15, 16). This difference can be attributed to variations in formal education and exposure to emergency care training. Doctors typically undergo more extensive and specialized medical education, including in-depth training in emergency medicine and critical care, while paramedics and nurses may have less comprehensive exposure to pre-hospital care knowledge areas. Healthcare providers in private hospitals had significantly higher knowledge scores compared to those in public hospitals, likely due to stricter adherence to guidelines, regular drills, effective monitoring, and better training opportunities and resource availability in private institutions. Similarly, providers working in hub hospitals exhibited higher knowledge scores than those in satellite hospitals, highlighting disparities in training, exposure, and resources between these settings.

Attitude scores also varied significantly based on workplace, with providers in private hospitals reporting higher median scores than those in public hospitals. This may be attributed to better exposure to emergency response protocols and more rigorous training programs in private institutions. However, unlike knowledge, attitude scores did not vary significantly by gender, profession, or years of experience, suggesting a widespread recognition of the value of pre-hospital care across these demographic factors. Interestingly, regional disparities in attitude scores were observed, with providers in Bagmati and Karnali provinces demonstrating the highest scores, while those in Lumbini province had the lowest. This variation may reflect differences in training programs, resource availability, and policy implementation across provinces.

### Implications for policy and practice

The findings of this study are consistent with global evidence on pre-hospital care challenges in LMICs. The lack of trained personnel and inadequate infrastructure reported in this study mirrors findings from Ethiopia (1) and Sri Lanka (12). Similarly, the emphasis on inter-agency collaboration and public-private partnerships aligns with recommendations from the World Health Organization (WHO) for strengthening emergency care systems in resource-limited settings (18, 19). However, the study also highlights unique challenges specific to Nepal, such as its geographical diversity and decentralized governance structure, which complicate the implementation of pre-hospital care policies. These findings underscore the need for context-specific solutions tailored to Nepal’s unique healthcare landscape.

### Strengths and limitations

Strengths:

- The study provides a comprehensive assessment of the knowledge, attitudes, and practices of healthcare providers in pre-hospital care in Nepal.
- The use of a systematic randomization technique ensures a representative sample across different regions and types of hospitals.
  Limitations:
- The study relies on self-reported data, which may be subject to social desirability bias.
- The cross-sectional design limits the ability to infer causality between demographic factors and KAP levels.
- The study focuses on healthcare providers in hospitals and may not be representative of those working in other settings.

## Conclusion

This study reveals that although healthcare providers in Nepal exhibit good knowledge and positive attitudes toward pre-hospital care, only 25% demonstrate good practice—highlighting a substantial gap between awareness and practical implementation. Disparities across gender, profession, and workplace suggest unequal access to training and resources, emphasizing the need for targeted interventions such as hands-on training and continuous professional development. Strengthening pre-hospital care in Nepal will require coordinated policy reforms, improved infrastructure, and enhanced collaboration among stakeholders. Addressing these gaps is critical to building a resilient emergency care system and improving patient outcomes nationwide.

## Data Availability

The raw data supporting the conclusions of this article will be made available by the authors, without undue reservation.

## References

[1] Abate H Mekonnen C. Knowledge, practice, and associated factors of nurses in pre-hospital emergency care at a tertiary care teaching hospital. Emerg Med. (2020) 12:459–69. doi: 10.2147/OAEM.S290074 PMC7781023 33408536

[2] Kumar AL Singh M Kapoor D. Prehospital trauma care services in developing countries. Anaesth Pain Intensive Care. (2013) 17:65–70.

[3] Bhattarai HK Bhusal S Barone-Adesi F Hubloue I. Prehospital emergency care in low- and middle-income countries: a systematic review. Prehosp Disaster Med. (2023) 38:495–512. doi: 10.1017/S1049023X23006088, PMID: 37492946 PMC10445116

[4] Wong EG Gupta S Deckelbaum DL Razek T Kushner AL. Prioritizing injury care: a review of trauma capacity in low and middle-income countries. J Surg Res. (2015) 193:217–22. doi: 10.1016/j.jss.2014.08.055, PMID: 25277355

[5] Wilson N Cunningham A Coleman E Patterson C Nnajiuba H Guerrero A. Challenges facing a new prehospital care service in the developing world: the Nepali ambulance service (NAS). Scand J Trauma Resusc Emerg Med. (2013) 21:1. doi: 10.1186/1757-7241-21-S1-S13

[6] Shrestha L Adhikari B Bajracharya M Aryal N Rajbhandari A Shrestha S. Triage processes in primary, secondary, and tertiary health care facilities in the Kathmandu Valley, Nepal: a mixed-methods study. BMC Emerg Med. (2024) 24:1–13. doi: 10.1186/s12873-024-01139-y PMC11590471 39587479

[7] Mehmood Amber Rowther AA Kobusingye O Hyder A. Assessment of pre-hospital emergency medical services in low-income settings using a health systems approach. Int J Emerg Med. (2018) 11:1–10. doi: 10.1186/s12245-018-0207-6 PMC6326122 31179939

[8] Kironji AG Hodkinson P De Ramirez SS Anest TL Razzak J. Identifying barriers for out of hospital emergency care in low and low-middle income countries: a systematic review. BMC Health Serv Res. (2018) 18:291. doi: 10.1186/s12913-018-3091-0 29673360 PMC5907770

[9] The World Health Organization. Integrated emergency, critical and operative care for universal health coverage and protection from health emergencies. Seventy-Fifth World Heal Assem. (2023) 13:1–6.

[10] Bolarinwa OA. Principles and methods of validity and reliability testing of questionnaires used in social and health science researches. Niger Postgrad Med J. (2015) 22:195–201. doi: 10.4103/1117-1936.173959, PMID: 26776330

[11] Chittaranjan A. The inconvenient truth about convenience and purposive samples. Indian J Psychol Med. (2021) 43:86–8. doi: 10.1177/0253717620977000, PMID: 34349313 PMC8295573

[12] Nandasena G Abeysena C. Knowledge, attitudes and skills of doctors, nurses and emergency medical technicians in pre-hospital care and emergency medicine who accompany patients in ambulances which arrive at the National Hospital of Sri Lanka. Int J Clin Anesth Res. (2018) 2:038–43. doi: 10.29328/journal.ijcar.1001010

[13] Banstola A Gautam P Smart G Sherpa F Joshi SK Mytton J. Prehospital emergency care for trauma victims in Nepal: a mixed-methods study. *Injury Prevention* (2024) 1:89–107. doi: 10.3310/TMTG2437 40014747

[14] Suryanto Plummer V Boyle M. Knowledge, attitude, and practice of ambulance nurses in prehospital care in Malang, Indonesia. Australas Emerg Care. (2018) 21:8–12. doi: 10.1016/j.auec.2017.12.001 30998865

[15] Sandeep Kumar SK Agarwal AK Akshay Kumar AK Agrawal GG Sushant Chaudhary SC Varsha Dwivedi VD. A study of knowledge, attitude and practice of hospital consultants, resident doctors and private practitioners with regard to pre-hospital and emergency care in Lucknow. *Indian Journal of Surgery* (2008) 70:14–18. doi: 10.1007/s12262-008-0003-2 PMC3452590 23133009

[16] Roshana S Batajoo KH Piryani R Sharma MW. Basic life support: knowledge and attitude of medical/paramedical professionals (2012) 3:141–5. doi: 10.5847/wjem.j.issn.1920-8642.2012.02.011 PMC4129799 25215053

[17] Chaudhary GP Sah K Malla J Das N Chaudhary S Chaudhary I. Knowledge regarding basic life support among health Care Workers of the Hospital of Nepal. J Healthc Eng. (2023) 2023:9936114. doi: 10.1155/2023/9936114, PMID: 36644299 PMC9836805

[18] Wang HE Mann NC Jacobson KE Ms MD Mears G Smyrski K. National characteristics of emergency medical services responses in the United States. Prehosp Emerg Care. (2013) 17:8–14. doi: 10.3109/10903127.2012.722178, PMID: 23072355

[19] WHO. Strengthening pre-hospital care through training of basic emergency medical technicians; (2021). Available Online at:https://www.who.int/nepal/news/detail/22-12-2021-strengthening-pre-hospital-care-through-training-of-basic-emergency-medical-technicians

